# St. George's Hospital and King Edward's Fund

**Published:** 1912-06-22

**Authors:** Savile Crossley, John G. Griffiths

**Affiliations:** Honorary Secretary.; Honorary Secretary.


### St. George's Hospital and King Edward's Fund.

WE take the following from the *Times* of June 10 and 12 :—

SIR,—We are not authorised to enter into a public discussion on the merits of the question at issue between St. George's Hospital and the King's Fund. Our only object in writing was to correct certain inaccuracies in the statement of the question which seemed calculated to prejudice the case to the detriment of the Fund. We note, however, that in their letters on this subject the hospital authorities claim to have evidence in their possession which gives a different result from that arrived at by the King's Fund. No information on this point has ever been addressed to the Fund. Any further evidence which the hospital may desire to submit to the Fund in support of its application for a grant would, of course, receive full consideration.—We are, Sir, your obedient servants,

SAVILE CROSSLEY,
JOHN G. GRIFFITHS,
June 5. Honorary Secretaries.

June 11. St. George's Hospital.

